# Cardiac and obstetric outcomes of pregnancies for women after cardiotoxic therapy in childhood: a single center observational study

**DOI:** 10.1186/s12885-023-10578-y

**Published:** 2023-02-02

**Authors:** Julius C. Heemelaar, Steffie Heemelaar, Svenja N. Hertel, J. Wouter Jukema, Marieke Sueters, Marloes Louwerens, M. Louisa Antoni

**Affiliations:** 1 Department of Cardiology, Leiden University Medical Center, Albinusdreef 2, 2333 ZA Leiden, the Netherlands; 2 Department of Obstetrics and Gynaecology, Leiden University Medical Center, Albinusdreef 2, 2333 ZA Leiden, The Netherlands; 3 Netherlands Heart Institute, Moreelsepark 1, 3511 EP Utrecht, The Netherlands; 4 Department of Internal Medicine, Leiden University Medical Center, Albinusdreef 2, 2333 ZA Leiden, The Netherlands

**Keywords:** Survivorship, Pregnancy, Heart failure, Anthracyclines, Cardiotoxicity, Echocardiography, Surveillance

## Abstract

**Background:**

Childhood cancer survivors (CCS) are at increased risk of cardiomyopathy during pregnancy if they have prior cardiotoxic exposure. Currently, there is no consensus on the necessity, timing and modality of cardiac monitoring during and after pregnancy. Therefore, we examined cardiac function using contemporary echocardiographic parameters during pregnancy in CCS with cardiotoxic treatment exposure, and we observed obstetric outcomes in CCS, including in women without previous cardiotoxic treatment exposure.

**Method:**

A single-center retrospective cohort study was conducted among 39 women enrolled in our institution’s cancer survivorship outpatient clinic. Information on potential cardiotoxic exposure in childhood, cancer diagnosis and outcomes of all pregnancies were collected through interviews and review of health records. Echocardiographic exams before and during pregnancy were retrospectively analyzed for left ventricular ejection fraction (LVEF) and global longitudinal strain (GLS) if available. The primary outcomes were (i) left ventricular dysfunction (LVD) during pregnancy, defined as LVEF < 50% or a decline of ≥ 10% in LVEF below normal (< 54%), and (ii) symptomatic heart failure (HF). Rate of obstetric and fetal complications was compared to the general population through the national perinatal registry (PERINED).

**Results:**

All pregnancies (91) of 39 women were included in this study. The most common malignancy was leukemia (N = 17, 43.6%). In 22 patients, echocardiograms were retrospectively analyzed. LVEF_baseline_ was 55.4 ± 1.2% and pre-existing subnormal LVEF was common (7/22, 31.8/%). The minimum value of LVEF during pregnancy was 3.8% lower than baseline (*p* = 0.002). LVD occurred in 9/22 (40.9%) patients and HF was not observed. When GLS was normal at baseline (< -18.0%; N = 12), none of the women developed LVD. Nine of out ten women with abnormal GLS at baseline developed LVD later in pregnancy. In our cohort, the obstetric outcomes seemed comparable with the general population unless patients underwent abdominal irradiation (N = 5), where high rates of preterm birth (only 5/18 born at term) and miscarriage (6/18 pregnancies) were observed.

**Conclusion:**

Our study suggests that women with prior cardiotoxic treatment have a low risk of LVD during pregnancy if GLS at baseline was normal. Pregnancy outcomes are similar to the healthy population except when patients underwent abdominal irradiation.

**Supplementary Information:**

The online version contains supplementary material available at 10.1186/s12885-023-10578-y.

## Background

Childhood cancer survivors (CCS) are confronted with a myriad of long term sequalae of their former treatment as they get older [1, 2]. These treatments include various forms of radiotherapy - total body irradiation (TBI), mantle field irradiation -, chemotherapy and hematopoietic stem cell transplantation (HSCT). The potential problems with reproductive health are a major concern for female cancer survivors, because having children is an important determinant of quality of life in this population [3]. While there are increased rates of gonadal dysfunction and infertility, it has been shown by several large studies that, for those who are able to conceive, overall obstetric outcomes are comparable to the general population [4, 5]. The exception are CCS who have been exposed to radiation therapy involving the uterus (e.g. TBI), who are at increased risk for miscarriage, preterm birth and low birthweight [4].

To diagnose and treat adverse late effects of cancer treatments, CCS are frequently enrolled in late effects out-patient clinics where they are periodically and systematically screened for a broad range of conditions including heart disease [6, 7]. Also patients who are treated at childhood or adolescence with chemotherapy and/or hematopoietic stem cell transplantation for non-malignant conditions such as severe aplastic anemia and thalassemia are enrolled at this clinic, because of increased rates of long-term cardiac and pulmonary toxicity [8, 9]. This screening is of paramount importance because prior cardiotoxic treatments (e.g. anthracyclines or thoracic radiotherapy) can cause subclinical cardiac damage that can manifest as left ventricular dysfunction (LVD) and heart failure (HF) during pregnancy or later in life. Furthermore, pre-existing LVD is a major risk factor for decline in cardiac function during pregnancy with a nearly 50-times increased risk compared to CCS without pre-existing LVD [10].

However, currently there is no consensus regarding necessity, timing and modality/parameter of cardiac monitoring during and after pregnancy [4]. This is a result of lack of robust data on changes of left ventricular function during pregnancy in this population and most studies utilized outdated parameters for cardiac monitoring (e.g. fractional shortening).

Therefore, we aimed to examine modern echocardiographic parameters of cardiac function throughout pregnancy and pregnancy outcomes in a cohort of women enrolled in the late effects out-patient clinic at the Leiden University Medical Center.

## Methods

A single-center retrospective cohort study was conducted at the late effects out-patient clinic in the department of Internal Medicine at Leiden University Medical Center in The Netherlands. This study was approved by the departmental institutional review board (study-ID: W2021.012) and executed according to the declaration of Helsinki.

### Setting

All CCS or children who received chemotherapy and/or hematopoietic stem cell transplantation are invited to and followed up at the late-effects out-patient clinic 5 years after completing treatment from the age of 18 years onwards, in accordance with the Dutch national LATER-protocol [7]. Detailed history of cancer diagnosis and treatment, cardiovascular risk factors including all forms of treatment, signs and symptoms are collected and routinely updated. Between every three to five years an echocardiogram is performed to assess myocardial function in survivors that received high-risk cardiotoxic cancer treatments. In pregnant women who received prior high-risk cardiotoxic cancer treatment, additional echocardiograms are made in the 1st trimester (T_1_) and 2nd to 3rd trimester (T_23_). If women developed LVD or HF during pregnancy, a post-partum (PP) echocardiogram was performed.

### Study participants

For this study all women followed up between founding of the clinic in 2008 and May 2021 were screened for pregnancy. Women were eligible for inclusion if they had been pregnant at least once. All pregnancies of women were recorded. Patients were not included if they were lost to follow up or did not consent to the study.

### Procedures

Potentially eligible patients were contacted by telephone between January and May 2022. When informed consent was provided, two investigators (JCH and SNH) collected data on all pregnancies from the last outpatient clinic visit before the pregnancy – the baseline visit – up to one year after the final pregnancy. If pregnancy related care was provided in another health institution, obstetric files were retrieved from these institutions. Echocardiograms were retrospectively analyzed from (1) the last clinic visit before monitored pregnancy (T_BL_) (2) first trimester (T_1_) and (3) the second or third trimester (T_23_). Echocardiograms of the first monitored pregnancy were analyzed if at least T_BL_ and T_1_/T_23_ were available. In patients with LVD or HF during pregnancy echocardiograms up to one year post-partum were also analyzed (T_post_).

### Variables

The following parameters were analyzed: cancer diagnosis and treatment – including cumulative anthracycline and radiotherapy dosages -, family history, cardiovascular risk factors, medication use before and during pregnancy, duration of pregnancy, mode of birth, obstetric complications, fetal and neonatal outcome. Developmental problems in the first year after birth were ascertained on reports of the mother. A HF risk score designed for childhood cancer survivors was calculated for all patients [11].

The parameters of interest were left ventricular end-systolic and end-diastolic volumes (LVESV, LVEDV), and left ventricular functional markers – left ventricular ejection fraction (LVEF) and speckle-tracking derived global longitudinal strain (GLS). GLS is a modern marker that can detect subclinical LVD and is increasingly used in cardio-oncology, due to the capability to predict future LV functional deterioration [12].

LVD was defined as LVEF < 50% during pregnancy or decline of ≥ 10% in LVEF below normal. For women the cut-off value for normal LVEF is 54% [13]. Abnormal GLS was defined as < (-) 18% [14]. HF was defined as symptoms of shortness of breath in combination with bibasilar crackles during auscultation or bibasilar pleural fluid on an X-ray and/or peripheral pitting oedema.

A viable pregnancy was defined as a pregnancy with a duration of 24 weeks and 0 days or more. Intrauterine fetal demise was defined as fetal death after 16 weeks of gestation. Postpartum hemorrhage was defined as blood loss of more than 1000 mL. Hypertensive and other disorders during pregnancy were defined according to the national guidelines [15, 16]. Premature birth was defined as birth before 37 weeks and 0 days of gestation, small for gestational age as birthweight < 10th percentile, low birthweight as birthweight < 2500 gram and neonatal death as death within 28 days after birth.

### Statistical analysis

Continuous variables are presented as mean ± standard deviation, or median with first and third quartile (Q1-Q3) depending on variable-distribution. Normality of distribution was assessed graphically and by Kolmogorov-Smirnov test. Categorical data are presented as frequencies and percentages.

Pregnancy outcomes of the included women were compared with pregnancy outcomes of the general Dutch population, using data from the national perinatal registry (PERINED) [17]. Echocardiographic parameters during and after pregnancy were compared to baseline values by paired Student *t*-test and Wilcoxon signed-rank test. To describe the association between cardiotoxicity risk and subsequent functional deterioration during pregnancy, baseline clinical and echocardiographic parameters and incident LVD were plotted.

Missing data was accounted for by available case analyses. A comparison of baseline characteristics was made between patients with and without echocardiographic monitoring during pregnancy to account for possible selection bias. Stata® version 16.1 was used for all analyses (StataCorp LLC, College Station, Texas, USA).

## Results

**Fig. 1:**
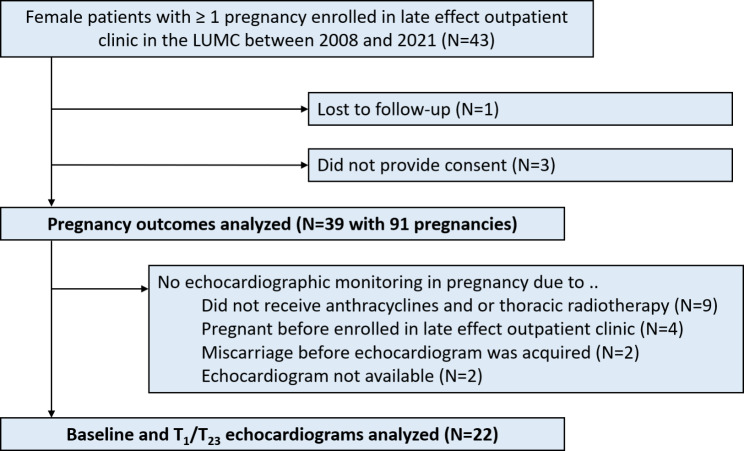
STROBE diagram with patient selection process GA = gestational age; T1/T23 = 1st trimester / 2nd or 3rd Trimester

Figure 1 summarizes the patient selection process. In total, in 39 out of 43 screened women all pregnancy related records and pregnancy outcomes could be obtained. In 22 of the 39 patients (56.4%), echocardiograms at baseline and during pregnancy were available for analysis. The most common reasons to not monitor patients with an echocardiogram were: no high-risk cardiotoxic treatments (N = 9), or the patient was pregnant before enrollment in the late effect outpatient clinic (N = 4). Baseline characteristics of the study population are shown in Table 1. Baseline characteristics of women with and without echocardiographic monitoring are compared in Supplemental Table S1.

**Table 1:** Baseline characteristics of study population

	**Total (N = 39)**
Index diagnosis	
Age at cancer diagnosis, years	7.3 (3.7–12.1)
Age at end first pregnancy, years	27.0 (23.0–29.0)
Index diagnosis	
Leukemia	17 (43.6%)
Lymphoma	5 (12.8%)
Sarcoma	8 (20.5%)
Aplastic anemia	6 (15.4%)
Homozygous beta-thalassemia	2 (5.1%)
Wilms’ tumor	1 (2.6%)
Treatment	
Doxorubicin treatment	29 (74.4%)
Equivalent cumulative dosage, mg/m^2^	270 (168–356)
Antimetabolites	15 (38.5%)
Alkylating agents	35 (89.7%)
Platinum-based agents	4 (10.3%)
Methotrexate	19 (48.7%)
Radiotherapy, any	11 (28.2%)
Total body irradiation	4 (10.3)
Thoracic radiotherapy	7 (17.9%)
Dosage, gray	12 (5–36)
Abdominal radiotherapy	5 (12.8%)
Brain radiotherapy	7 (17.9%)
Neck radiotherapy	10 (25.6%)
Other risk factors	
Heart failure risk score*	5 (4–7)
Low risk (< 3)	4 (10.3%)
Medium risk (3–5)	19 (48.7%)
High risk (> 5)	16 (41.0%)
History of cardiotoxicity	5 (12.8%)
History of heart failure	1 (2.6%)
Smoking status prior to first pregnancy	
Never smoked	32 (82.1%)
Stopped smoking	6 (15.4%)
Active smoker	1 (2.6%)
Family history of premature cardiovascular disease	7 (17.9%)
Betablocker use up to 1st trimester of 1st pregnancy	3 (7.7%)

Data are presented as median (Q1-Q3) for continuous measures, and N (%) for categorical measures. *based on standard model of Chow et al. (11)

Among the 39 included patients the most common index diagnosis was leukemia (N = 17, 43.6%) and sarcoma (N = 8, 20.5%). Eight patients had a hematological condition such as aplastic anemia (N = 6; 15.4%) for which they were treated with chemotherapy and/or hematopoietic stem cell transplantation at childhood.

The cardiotoxicity risk in the study population was substantial. Three quarters of patients underwent thoracic radiotherapy and/or anthracycline containing chemotherapy. The median HF risk score was 5 (Q1-Q3: 4–7) and 16 patients (41.0%) had a score of > 5, which corresponds to a relative risk of 33 and higher for HF later in life compared to siblings without prior cardiotoxic treatment [11].

### Echocardiography and heart failure during pregnancy

**Fig. 2:**
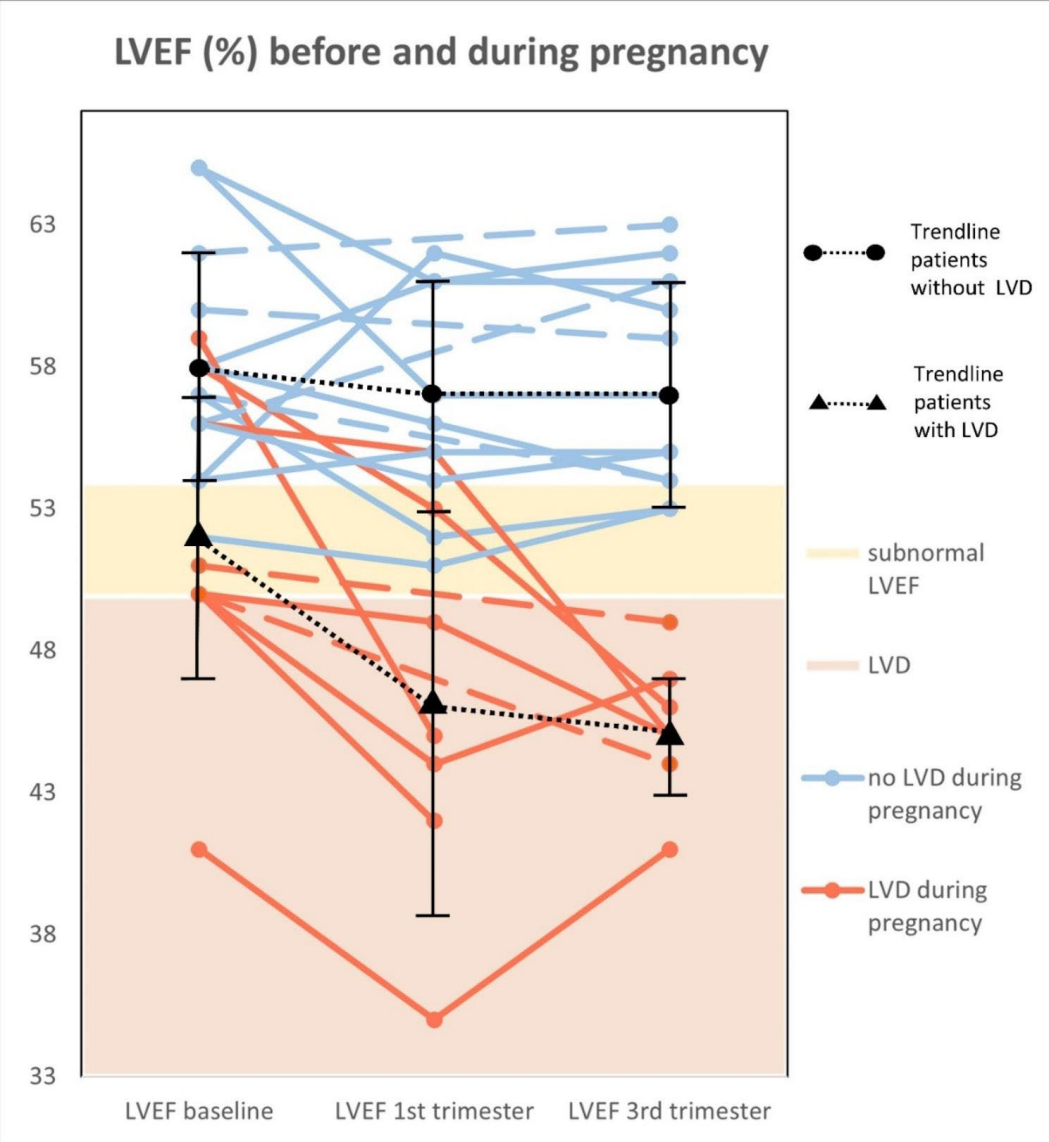
Spaghetti plot of left ventricular function before and during pregnancy The lines represent cases that develop LVD (red) and do not develop LVD (blue). Dotted lines indicate that a measurement in the 1st trimester is missing. The summary black summary lines represent mean ± SD

The echocardiograms of 22 patients during pregnancy were retrospectively analyzed. In half of the cases the first pregnancy was monitored (N = 11). In eight women the second pregnancy and in three women three or more pregnancies were monitored. In 9/22 women the criteria for LVD were met (40.9%). Clinical HF was not observed during pregnancy. An overview of the progression left ventricular parameters is shown in Table 2; Fig. 2.

**Table 2:** Echocardiographic parameters before and during pregnancy and recovery

Parameter / GA	Baseline (n = 22) − 15 ± 10 months	1st trimester (n = 16) 11 ± 3 weeks	3rd trimester (n = 20) 30 ± 4 weeks
LVEDV (ml)	94 ± 19	105 ± 23*	107 ± 26*
LVESV (ml)	42 ± 11	50 ± 16*	51 ± 16*
LVEF (%)	55 ± 6	52 ± 7*	53 ± 7*
GLS (%)	(-)18.0 ± 2.4	(-)17.5 ± 3.2	(-)17.3 ± 8.4*

*p < 0.05 vs. baseline

GA = gestational age; LVEDV = left ventricular end-diastolic volume; LVESV = left ventricular end-systolic volume; LVEF = left ventricular ejection fraction; GLS = global longitudinal strain

All variables are displayed as mean ± SD

At baseline the mean LVEF was 55.4% ± 1.2%. A pre-existing subnormal LVEF was common: 7/22 women (31.8%) had a LVEF below 54% of which one patient had an LVEF below 50%. Compared to baseline, the LVEF decreased in T_1_ (mean difference [MD] = -2.8%; *p* = 0.037) and remained reduced in T_23_ (mean difference = -2.3%, *p* = 0.045). Overall, the minimum value of LVEF during pregnancy was 3.8% lower compared to baseline (*p* = 0.002). GLS was mildly - though significantly - reduced in T_23_ compared to baseline (MD = 0.81, *p =* 0.027).

In women who met the criteria for LVD, LV function did not always fully recover. Monitoring echocardiograms post-partum still showed a significant reduction compared to baseline within a year (median 50% vs. 46%; *p* = 0.031) and non-significant reduction after more than 1 year post-partum (50% vs. 48%; *p* = 0.06*)*. Median baseline LVEF was 50 (50–56%) with a median 5% drop in LVEF during pregnancy.

### Description of baseline echocardiographic parameters and incident LVD

The conventional method of echocardiographic LV function assessment, LVEF, was well able to predict LVD during pregnancy if reduced: of seven women with subnormal LVEF six developed LVD. Furthermore, all women with a history of LVD or HF (N = 3) experienced LVD during pregnancy. In the 15 women with a normal LVEF, 3 women still developed LVD.

**Fig. 3:**
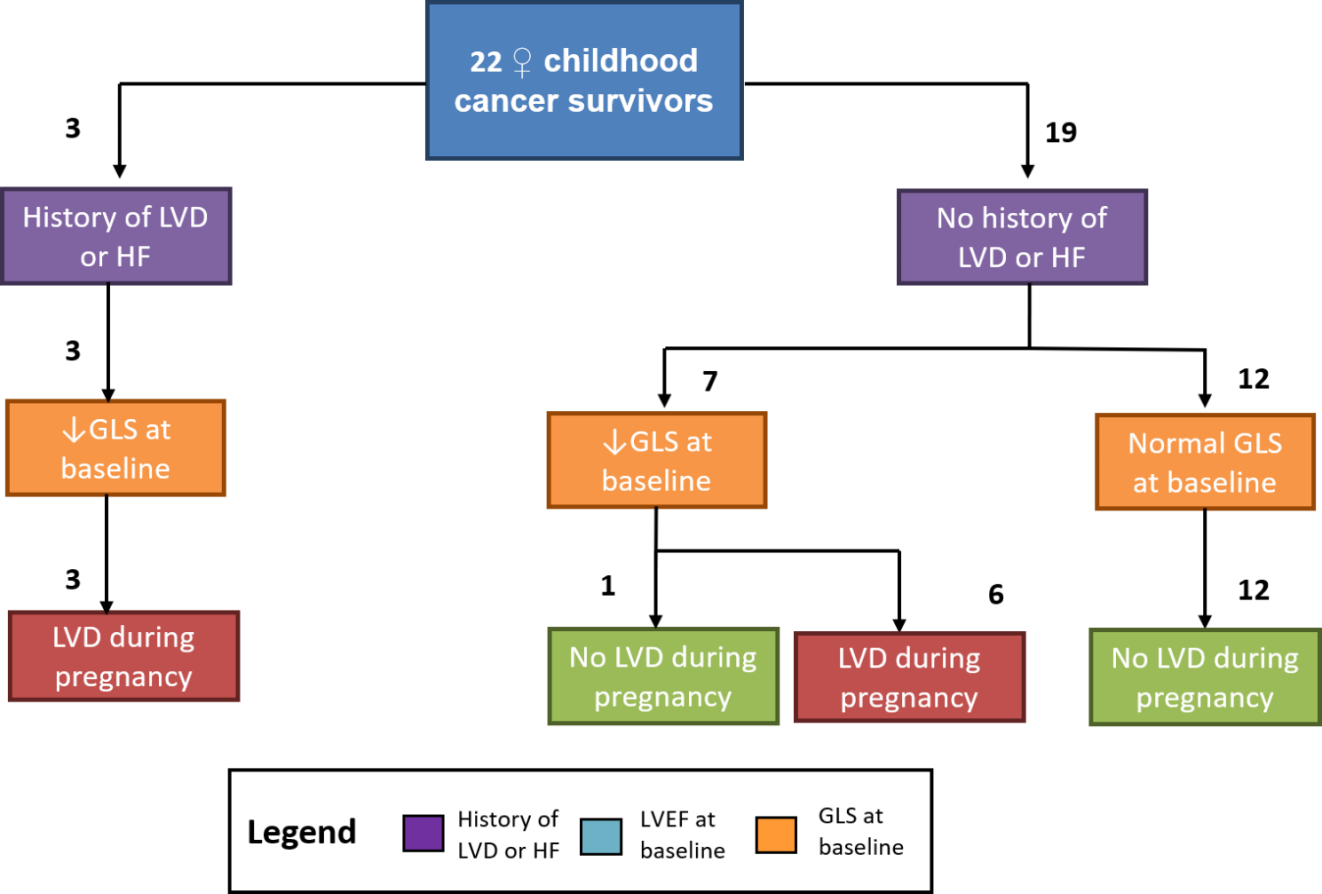
Flowchart of incident left ventricular dysfunction stratified by past cardiotoxicity and baseline GLS.

On the contrary, GLS showed good ability to predict both LVD and absence of LVD: All 12 women with a normal GLS with baseline did not develop LVD during pregnancy. In 10 cases where baseline GLS was reduced, 9 cases did indeed develop LVD during pregnancy. When a history of LVD or HF was added to the algorithm incident LVD was correctly classified in 21/22 cases (95.5%), see Fig. 3.

### Comparison LVEF throughout pregnancy to previous studies in normal population

When we compared the course of LVEF in our population to previous studies in a normal population, we found that the baseline LVEF was lower: 55% vs. 61–68%, respectively [18–22]; see Table 3. Furthermore, we found that the physiological decline in LVEF was observed earlier in pregnancy– i.e. in the first trimester compared to the third trimester.

**Table 3:** Overview of LVEF in our study population compared to different studies in women without prior cancer treatment

LVEF (%)	Baseline/controls	1st trimester	3rd trimester
This study n = 22	55 ± 6	52 ± 7	53 ± 7
C.M. Schannwell et al. (2002) n = 46	61 ± 4	61 ± 2	55 ± 5
K. Melchiorre et al. (2016) n = 559	65 [55–69] n = 50	61 [56–66] n = 109	60 [54–65] n = 102
J. Cong et al. (2015) n = 68	68 ± 5 n = 30	68 ± 5	65 ± 4
M.A.M. Kampman et al. (2016) n = 49		62 ± 6	60 ± 6
Y. Kimura et al. (2019) n = 397			63 ± 4

All variables are displayed as mean ± SD or as median [iqr]

### Pregnancy outcomes

The 39 included women had 91 pregnancies available for data analysis, see Table 4. Six pregnancies in four women occurred after fertility treatment. No spontaneous pregnancies among these four women occurred. Except for one, they were all pregnancies after oocyte donation. Of all pregnancies, 14 (15.4%) ended in a spontaneous miscarriages in the first trimester, 3 (3.3%) in a intrauterine fetal demise and one woman (1.1%) had an abortion. The remaining 73 pregnancies reached a viable gestation of > 24 + 0 weeks. Eight pregnancies were complicated by pregnancy induced hypertension and two with pre-eclampsia. There were no neonatal deaths. Eight neonates (8/73, 11.0%) were born < 37 weeks. One infant died four months after birth due to a congenital autosomal recessive disorder of which both the oocyt donor and the father appeared to be carrier. The other neonates appeared to have develop normally at one year of age.

**Table 4:** Pregnancy outcomes

	Total (%*)	General population†
# of pregnancies (N = 39 women)		
1	8 (20.5)	
2	18 (46.2)	
3	9 (23.1)	
4	2 (5.1)	
5	1 (2.6)	
7	1 (2.6)	
Outcome stratified by gestational age, weeks (N = 91 pregnancies)		
Live birth > 37 + 0	65 (71.4)	
Live birth 32 + 0 to 36 + 6	4 (4.4)	
Live birth 28 + 0 to 31 + 6	1 (1.1)	
Live birth 24 + 0 to 27 + 6	3 (3.3)	
Miscarriage < 12 + 0	14 (15.4)	
Miscarriage 12 + 0 to 15 + 6	0 (0.0)	
Intrauterine fetal demise > 16 + 0 to 23 + 6	3 (3.3)	
Abortion	1 (1.1)	
Mode of birth (N = 73 live births)		
Spontaneous vaginal birth	55 (75.3).	P = 74.4%
Assisted vaginal birth	7 (9.6).	P = 7.0%
Caesarean section	11 (15.1).	P = 15.2%
Maternal outcome (N = 73 live births)		
Hypertensive disorders during pregnancy		
None	63 (86.3)	
Pregnancy induced hypertension	8 (11.0)	P = 5.7%
Pre-eclampsia	2 (2.7).	P = 0.5%
Diabetes	0 (0.0)	P = 4.0%
Postpartum hemorrhage > 1L	1 (1.4)	P = 6.5%
Endometritis postpartum, treated medically	1 (1.4)	P = 1.7%
Manual removal of retained placenta	3 (4.1)	
Laparotomy for spontaneous lesion of uterine serosa	1 (1.1)	
Fetal outcome (N = 73 live births)		
Apgar < 7 at 5 minutes post-partum	2 (2.7)	P = 2.0%
Birthweight < p10 for gestational age	10 (13.7)	P = 10%
Preterm birth	8 (11.0)	P = 6.9%
Intra-uterine fetal death	0	P = 0.3%
Neonatal death‡	0	P = 0.2
Congenital disease§	1 (1.4%)	P = 1.0%

* denominator is reported within brackets per category in left column. † Source: Dutch national perinatal registry (PERINED). ‡ defined as: death within 28 days post-partum. § Infant died four months after birth

**Fig. 4:**
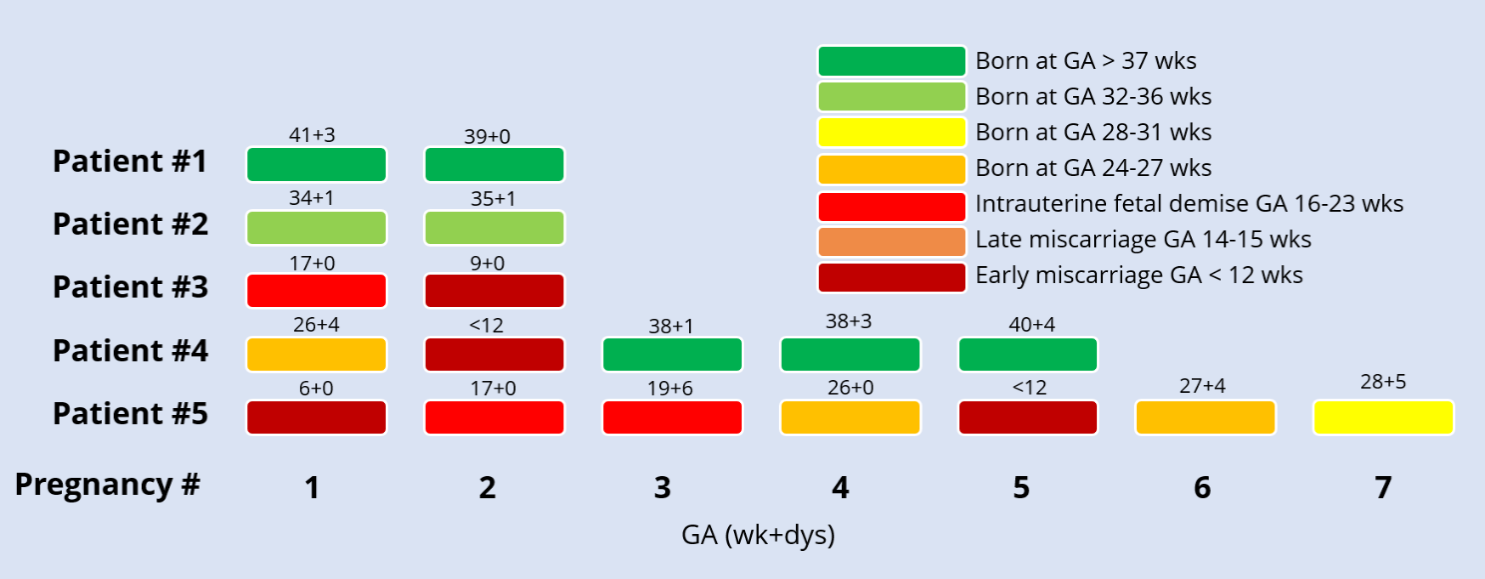
Graphic representation of pregnancy outcomes and gestational age in women with prior abdominal radiotherapy GA = gestational age; RT = radiotherapy; TBI = total body irradiation; wk + dys = weeks and days

Figure 4 represents outcomes of each of the pregnancies of five women whom were exposed to abdominal radiotherapy. In total these women experienced 18 pregnancies. In the first woman, the uterus was not in the treatment field and she gave birth to two at term neonates. Of the remaining 16 pregnancies 7 of these resulted in miscarriage and 5 were born at term. Of the 11 pregnancies that reached a viable gestational age, 2 neonates were small for their gestational age. Figure 3 shows the gestational age increases in subsequent pregnancies for the second, fourth and fifth women. The last pregnancy of the fifth woman ended with a severe complication as the woman presented with acute abdominal pain and free fluid abdominally. On laparoscopy a spontaneous lesion of the uterine serosa was found with active bleeding. A caesarean section was performed and the uterus was repaired. The woman lost 1 L of blood. Both woman and neonate recovered well.

## Discussion

We conducted a retrospective observational study among a cohort of pregnant women with prior cancer treatment and found that there was no LVD during pregnancy if LVEF and GLS at baseline were normal. To our knowledge this is the first study to examine the role of GLS in cardiotoxicity monitoring of pregnant women. Secondly, we found that overall pregnancy outcomes were comparable with the general population. The only exception seems to be that patients who underwent abdominal radiotherapy, experienced higher rates of preterm birth and miscarriage.

### Pregnancy-associated cardiomyopathy

An overall decline in LVEF during pregnancy was observed compared to baseline. Studies in healthy women show a similar decline in LVEF during pregnancy. The difference, however, is that the baseline LVEF in CCS was substantially lower compared to healthy women and the decline in LVEF was observed earlier in pregnancy compared to the normal population [18–22].

One could speculate that patients who received cardiotoxic treatment have less compensating cardiac ability in case of volume expansion due to pregnancy. In normal pregnancies, most hemodynamic adaptation occurs in T_1_ (until 20 weeks) [23]. Cardiotoxic treatments cause subclinical myocardial dysfunction through various pathophysiological mechanisms, such as interstitial fibrosis caused by thoracic radiotherapy and direct cardiomyocyte damage caused by anthracyclines. As a result of the subclinical myocardial dysfunction, LVEF may already start to decline at an earlier stage due to volume expansion compared to patients who did not receive cardiotoxic treatments. This could explain why in our vulnerable patient population LVEF reduction already occurred in T_1_ and not in T_23_ as has been reported in the general population.

HF was not observed in our cohort, which confirms the conclusion of a meta-analysis by Nolan and colleagues which states that HF during pregnancy in this population is rare [10]. They summarized all six existing cohort studies cornering this topic: an incidence of 1.7% of LVD and HF was observed and when a history of LVD or HF was present, these patients had an odds ratio of 47 on experiencing LVD during pregnancy. The observed cumulative incidence of LVD and HF is markedly lower compared to our study. The 2 most likely reasons are (1) routine echocardiograms during pregnancy were rarely performed and outdated parameters of LV function which are less sensitive to detect LVD were utilized. (2) By far the largest study in the meta-analysis was a study by Hines et al. where the high-risk cardiotoxic exposure was lower compared to our study: 57% vs. 100% of monitored patients had a history doxorubicin use, respectively.

To our knowledge this is the first study to assess GLS in pregnancy in recipients of cardiotoxic treatments during childhood. The results are promising, because in all cases GLS identified who did not develop LVD during pregnancy. This finding should prompt similar investigations in larger and multicenter datasets, because this could help select patients that would not have to be burdened with additional monitoring during pregnancy.

### Pregnancy outcomes

Reproductive health is of great importance to female cancer survivors. We observed low rates of obstetric complications among the women who were not treated with abdominal radiotherapy, and these rates were comparable to those reported in the general Dutch population [17]. However, women who were treated with abdominal radiotherapy – including TBI - do face higher risk of miscarriage, preterm birth and low birthweight, which was also observed in our cohort [24].

Several publications have described the impact of abdominal radiotherapy and poor pregnancy outcomes [4, 5, 25–28]. The most important determinants of pregnancy outcome is radiotherapy type and cumulative dose. In our selected cohort of women who were able to become pregnant, one woman who underwent abdominal radiotherapy where the uterus was outside the radiation field had two healthy at term born babies.

The pathophysiology of poor pregnancy outcomes in this population is assumed to be multifactorial. One of the main components is thought to be reduced uterine elasticity and volume as a result of myometrium fibrosis and impaired blood flow. In Fig. 3 may be observed that with subsequent pregnancies the gestational age seems to increase. One explanation could be that part of uterine elasticity and blood flow restores after a pregnancy. To our knowledge this observation has not been reported elsewhere. However, it has been reported that uterine volume seems to restore after a pregnancy [29]. In healthy women uterine volume is higher in gravidous women, compared to women who have never been pregnant [30]. When comparing radiotherapy exposed childhood cancer survivors and healthy controls, a significantly lower uterine volume was only identified among nulliparous radiotherapy exposed childhood cancer survivors, while after a pregnancy uterine size was comparable for both groups [29]. One woman with prior abdominal irradiation developed a spontaneous lesion of the uterine serosa, that was actively bleeding, in the latest of her seven pregnancies. To our knowledge, such a complication has not been reported elsewhere after abdominal radiotherapy. However, we did find two other case reports of a spontaneous uterine rupture in CCS with previous abdominal radiotherapy, also with an unscarred uterus [31, 32], which may suggest that women with previous abdominal radiotherapy are at risk for this rare but severe complication. For obstetricians it is important to be aware of this potentially lethal, however rare complication.

Currently there is no evidence for a safe dose of TBI, because adverse pregnancy outcomes have been reported in case reports and small cohort studies from all dose ranges [4]. In our study TBI was administered at low dosage (range: 4–12 Gy) and still pregnancy outcomes were poor. Therefore one could speculate that there is no safe dose of TBI with regards to reproductive health.

### Limitations

The main limitation of our study was the small sample size and that our study is a single center investigation. Therefore these results are foremost hypothesis generating. Furthermore, our study did not have a control group for both pregnancy outcomes and echocardiographic findings. However, we were able to compare the results with real-world data through the national perinatal registry [17].

A second key limitation was that not all patients were monitored with echocardiography – only those patients with cardiotoxic exposure. Fortunately, in the majority of cases, an echocardiogram could be obtained of the first pregnancy with sufficiently long gestational age i.e. in case of early miscarriage an echocardiogram was not performed. As this study only assessed pregnancy outcomes, our findings only concern female cancer survivors who were able to conceive.

## Conclusion

Our study suggests that women with prior cardiotoxic treatment have low risk of LVD during pregnancy if LVEF and GLS at baseline were normal. More investigation into GLS for risk stratification at time of pregnancy could assist to determine which patients would benefit from additional echocardiographic monitoring during pregnancy and, equally important, for which patients additional monitoring is not needed. Pregnancy outcomes are similar to the healthy population except when patients underwent TBI.

## Electronic supplementary material

Below is the link to the electronic supplementary material.

Supplementary Material 1

## Data Availability

All data generated or analyzed during this study are included in this published article and its supplementary information files.

## Declarations

### Trial registration

NA.

## List of abbreviations

CCS: Childhood cancer survivor(s)
HF: Heart failure
GA: Gestational age
GLS: Global longitudinal strain
LVD: Left ventricular dysfunction
LVEDV/LVESV: Left ventricular end-diastolic/end-systolic volume
LVEF: Left ventricular ejection fraction
T_1_/T_23_: First trimester / second to third trimester
TBI: Total body irradiation
