# Supplementary material for: Cardiac and obstetric outcomes of pregnancies for women after cardiotoxic therapy in childhood: a single center observational study
Source: BMC Cancer. 2023 Feb 2;23:115. doi: 10.1186/s12885-023-10578-y (PMC9893596; doi:10.1186/s12885-023-10578-y)
Supplement: Supplementary file 1 — Supplementary Material 1 [file 12885_2023_10578_MOESM1_ESM.docx]

Supplemental Table S1: Characteristics of group with echocardiographic monitoring versus no echocardiographic monitoring.

|  | Total (N=39) | Echo – (N=17) | Echo + (N=22) | *p*-value |
| --- | --- | --- | --- | --- |
| Age at cancer diagnosis | 7.3 (3.7-12.1) | 7.1 (4.8-11.2) | 9.2 (3.6-12.1) | 0.93 |
| Primary malignancy |  |  |  | 0.008 |
| Leukemia | 17 (43.6%) | 6 (35.3%) | 11 (50.0%) |  |
| Lymphoma | 5 (12.8%) | 1 (5.9%) | 4 (18.2%) |  |
| Sarcoma | 8 (20.5%) | 2 (11.8%) | 6 (27.3%) |  |
| Aplastic anemia &  homoyzygous beta-thalassemia | 8 (20.5%) | 8 (47.1%) | 0 (0.0%) |  |
| Wilm’s Tumor | 1 (2.6%) | 0 (0.0%) | 1 (4.5%) |  |
| Doxorubicin | 29 (74.4%) | 7 (41.2%) | 22 (100.0%) | <0.001 |
| Cumulative dose | 270 (168-356) | 300 (200-360) | 263 (162-356) | 0.61 |
| Radiotherapy, any | 11 (%) | 4 (%) | 7 (%) | 0.57 |
| Thoracic irradiation | 7 (17.9%) | 3 (17.6%) | 4 (18.2%) | 0.97 |
| Cumulative dose | 12 (5-36) | 12 (4-40) | 22 (6-36) | 1.00 |
| Heart Failure Risk Score | 5 (4-7) | 3 (3-5) | 6 (5-7) | <0.001 |
| low risk (<3) | 4 (10.3%) | 4 (23.5%) | 0 (0.0%) | 0.007 |
| med risk (3-5) | 19 (48.7%) | 10 (58.8%) | 9 (40.9%) |  |
| high risk (>5) | 16 (41.0%) | 3 (17.6%) | 13 (59.1%) |  |
| History of cardiotoxicity | 5 (12.8%) | 2 (11.8%) | 3 (13.6%) | 0.86 |
| History of heart failure | 1 (2.6%) | 0 (0.0%) | 1 (4.5%) | 0.37 |
| Echo - : no echocardiographic monitoring during pregnancy; Echo + : echocardiographic monitoring during pregnancy. | | | | |
